# Exploring Lumbar Spine Posture and Movement in Sitting: A Comparison Between Laboratory and Real-World Measures

**DOI:** 10.3390/jcm14217518

**Published:** 2025-10-23

**Authors:** Mansour Abdullah Alshehri, Ryan Riddick, Manuela Besomi, Wolbert van den Hoorn, David M. Klyne, Paul W. Hodges

**Affiliations:** 1 School of Health and Rehabilitation Sciences, The University of Queensland, St. Lucia, QLD 4072, Australia; r.riddick@uq.edu.au (R.R.); m.besomimolina@uq.edu.au (M.B.); w.vandenhoorn@uq.edu.au (W.v.d.H.); d.klyne@uq.edu.au (D.M.K.); 2 Department of Medical Rehabilitation Sciences, Faculty of Applied Medical Sciences, Umm Al-Qura University, Mecca 24382, Saudi Arabia; 3 Physical Therapy Department, Universidad del Desarrollo, Av. Plaza 680, Las Condes 7610615, Chile; 4 School of Exercise and Nutrition Sciences, Queensland University of Technology, Kelvin Grove, QLD 4059, Australia

**Keywords:** lumbar spine, spine posture, spine alignment, postural control, sitting, real world

## Abstract

**Background/Objectives**: Sitting is linked to health problems, including back pain. Sitting posture is commonly measured in the laboratory, but it remains unclear how this relates to real-world spine posture. **Methods**: A cross-sectional study of pain-free adults conducted measurements in “laboratory” and “real-world” settings. Wearable motion sensors recorded lumbar spine angular orientation to compare lumbar spine flexion angle during sitting postures in between settings. Gaussian mixture models defined participant-specific modes and overall probability distributions of real-world sitting posture. Measures included periods of real-world sedentary/activity behaviours and trunk postural control (laboratory). **Results**: Laboratory measures of lumbar angle were more flexed during questionnaire (30.0°) than upright (19.8°) sitting. The angle in unstable sitting was intermediate (27.1°). Spine posture in unstable sitting correlated with real-world overall mean posture (r = 0.49–0.54) and most frequent mode (r = 0.47). Upright laboratory sitting posture correlated with real-world second most frequent mode (r = 0.54). Sitting less (r = 0.45) and walking more (r = 0.41) in the real world related to better balance performance and lumbar spine coordination. **Conclusions**: Spine posture in an unstable sitting laboratory task had the closest association with real-world sitting but does not replicate the diversity of spine postures adopted in real-world sitting. Wearable sensors are viable to study real-world postures.

## 1. Introduction

Time spent sitting is increasing and linked with public health concerns [1]. Although many studies consider when people sit, it is also important to consider how people sit. Some investigations of sitting have identified possible relationships between spine kinematics/kinetics and musculoskeletal problems [2,3,4,5]. For example, more end-range postures (e.g., kyphotic/slumped or lordotic posture) [6,7,8,9,10] and specific features of movement (e.g., poor balance/coordination or spinal stiffness) [5,11,12] are thought to mechanically load spinal tissues, with relevance for the development and/or persistence of low back pain (LBP) [7,10,13]. Although plausible, evidence of a causal relationship is scant [14,15]. A major barrier has been the reliance on laboratory approaches to assess spine posture and movement because measurement in the real world has been virtually impossible [16]. If and how laboratory measures reflect real-world behaviours are unclear.

Most laboratory-based analyses rely on measurements made at an instant in time when the participant is aware they are being observed. Posture measured in this way differs from measures made when they are unaware that posture is being evaluated [17,18]. Some studies report that spine curvature measured during sitting in the laboratory differs between individuals with and without LBP [8,9]. Other work has investigated postural behaviours in sitting, such as the evaluation of postural equilibrium and orientation of the spine while sitting on an unstable seat [19,20]. Balance is maintained during this task by finely coordinated hip and lumbar spine movements [20,21], which differ between individuals with and without LBP [5]. Unstable sitting is a dynamic task, and its success might depend on features such as spine posture. Whether spine posture influences performance during unstable sitting and whether this posture and/or performance relates to sitting in the real world are unknown. Whether typical physical activity affects performance during unstable sitting is also unknown.

Advances in wearable sensors enable detailed and long-term monitoring in naturalistic unconstrained settings [16,22] and might provide insights into biomechanical factors of LBP [23]. Sensors that integrate accelerometers, gyroscopes, and magnetometers can be used to estimate joint orientation [16,22]. Measures correlate with those obtained using optical motion capture systems in unstable sitting [24], standing [25], and walking [26].

This study aimed to investigate whether (1) sitting postures adopted in the laboratory relate to sitting postures measured with wearable sensors in the real world for 48 h; (2) posture in unstable sitting in the laboratory is more strongly associated with real-world postures than other laboratory sitting postures (e.g., instruction to sit upright); (3) balance performance or spine coordination during unstable sitting is better in people who are less sedentary; and (4) posture adopted in unstable sitting (laboratory) relates to the performance of this task.

## 2. Materials and Methods

### 2.1. Participants

This cross-sectional study involved 25 healthy pain-free participants (age: 30 ± 13 years; female/male: 13/12; body mass index [BMI]: 22.30 ± 2.95 kg/m^2^) recruited through advertisements around the university, local community, and social media. Participants were excluded if they were <18 or >75 years old, had a history of LBP and/or any major pain/injury within the past six months, a history of spinal surgery, current disease(s)/disorder(s) likely to impact findings including balance, or were currently/recently pregnant. Ethical approval was granted by the Institutional Human Research Ethics Committee. Participants provided informed consent.

### 2.2. Experimental Setup

Data were collected in the laboratory and real world using dorsaVi (dorsaVi Ltd., Melbourne, Australia) and activPAL (PAL Technologies Ltd., Glasgow, UK) wearable motion sensors. DorsaVi sensors were positioned over the lumbosacral junction (L5/S1); the thoracolumbar junction (T12/L1); and the anterior right mid-thigh. A sensor was placed on the back of the seat during the unstable sitting paradigm (laboratory session). The sampling frequency for all sensors was 100 Hz during laboratory testing and reduced to 20 Hz for the accelerometer and gyroscope and 10 Hz for the magnetometer during real-world testing to maximise battery life. For comparison, laboratory-based data were down-sampled to 20 Hz. This aligned the laboratory-based data with the 20 Hz real-world recordings, which were selected for battery and storage feasibility over 48 h, and is consistent with a prior spinal (lumbar) posture study that used a 20 Hz sampling rate in the laboratory with similar sensors (dorsaVi) [25], adequately capturing the predominantly low-frequency outcomes analysed. An activPAL sensor was also attached on the anterior right mid-thigh to provide contextual information in the real world by classifying periods of sitting, standing, stepping, lying, and seated transport. ActivPAL data were recorded at a sampling frequency of 10 Hz for both laboratory and real-world settings and were up-sampled to 20 Hz to match the accelerometer of the dorsaVi sensors.

### 2.3. Experimental Procedure

#### 2.3.1. Laboratory Testing

Three sitting tasks were included for the laboratory analysis:

- Upright sitting: participants were instructed to “sit normally” in an upright position on a stool without a backrest (10 s) with their arms crossed and knees flexed at 90°.
- Questionnaire sitting: participants were instructed to “sit as you prefer” on a stool while completing an online questionnaire (2–5 min) via their smartphone.
- Unstable sitting: participants maintained balance while sitting on an unstable surface [19,20]. Part of an aluminium hemisphere (undersurface of the seat; radius: 250 mm; height of seat: 195 mm) and an adjustable footplate to accommodate 90° knee flexion were attached to the seat. A safety bar was placed in case of balance loss. Participants maintain balance during trials with eyes open (EO unstable sitting) and closed (EC unstable sitting) and were instructed to “sit relaxed and upright as quietly as possible”. Six trials were performed (duration 60 s, rest ~60 s) in random order of three with EO and EC.

#### 2.3.2. Real-World Testing (48 h)

After the laboratory session, sensors were replaced with a second set covered with waterproof adhesive tape. The standardised sensor placement ensured comparability between measures. Participants were asked to go about their “daily life” for 48 h of continuous monitoring.

### 2.4. Data Processing

#### 2.4.1. Estimation of Spine Orientation

To reduce gyroscope bias, which affects the accuracy of estimation over longer periods [27], an automated detection algorithm identified when the sensors were not moving (below a threshold). Gyroscope bias was estimated for continuous stationary periods > 5 s, and a linear model estimated the time evolution of the bias, which was subtracted from the gyroscope signal.

Orientation of each dorsaVi sensor was estimated using a Gradient Descent Filter [28] with tuning parameters optimised for a sampling rate of 20 Hz across recorded tasks. The orientation filter outputs the quaternions for each sensor, and the lumbar spine curvature was defined using the data of both spine sensors by multiplying one sensor with the conjugated quaternion of the other and transforming the quaternions into Euler angles using ‘ZXY’ sequence of rotations [20]. Information about data synchronisation, segmentation, and exclusion is provided in Supplementary Material File S1.

#### 2.4.2. Modelling Distributions of Real-World Spinal Postures

**Figure 1:**
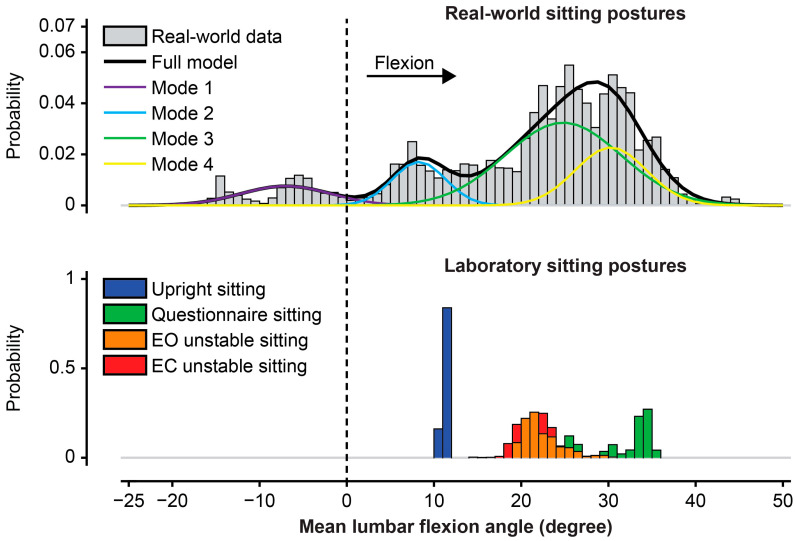
An example of the Gaussian Mixture Model (GMM) in one participant for estimating modes of sitting postures that are frequently adopted over 48 h in the real world. Graphs demonstrate the GMM modes (Mode 1 [purple], Mode 2 [Turquoise], Mode 3 [green], Mode 4 [yellow]) of adopted sitting postures in the “real world” and the different tested sitting postures in the “laboratory” (upright sitting, questionnaire sitting, eyes open [EO] unstable sitting, eyes closed [EC] unstable sitting) based on the mean lumbar flexion angle outcome. For the GMM modes, the greater the area under the curve, the more frequent (“relevance”) is the use of this posture mode. The “relevance” is a probability ranging from 0 to 1, representing how much of the total data is explained by that mode. In this example, mode 3 has the greatest area under the curve, which reflects the most frequent posture adopted in the real world for this participant. In the laboratory, the mean lumbar flexion angle adopted in both EO and EC unstable sitting is in the middle, more flexed than upright sitting but more extended than questionnaire sitting, suggesting a more neutral posture. A value of zero (dashed vertical line) represents a posture adopted by the participant during upright standing, with positive values indicating postures that are more flexed than standing.

Spine curvature in sitting varied over the 48 h of real-world recording. During individual bouts of sitting, there were periods with a Gaussian distribution of postures. There were also discrete shifts in posture between modes, both within bouts and across different bouts of sitting that were interrupted by standing and walking. To account for the distribution shape and make meaningful comparisons to sitting postures in the laboratory, we used Gaussian Mixture Models (GMMs) to estimate modes of lumbar spine sitting postures that were frequently adopted. Preliminary exploration of the data showed that using >4 modes often resulted in overfitting. To avoid overfitting, we used the Calinski–Harabasz criterion to evaluate the optimal number of modes, with up to 4 (Figure 1).

#### 2.4.3. Outcome Measures

The mean angle of the lumbar spine in the sagittal plane (relative orientation between L5/S1 and T12/L1 sensors) in sitting was calculated. A positive angle indicated a flexed lumbar spine posture relative to standing, whereas a negative angle indicated an extended posture. The reference for the mean lumbar angle in sitting (defined as zero) was the median lumbar angle across all periods of standing (laboratory and real world separately) [29]. The mean lumbar angle was calculated and compared for sitting postures adopted in the laboratory and the real world (identified using activPAL outputs of sitting periods). Variables were overall posture [overall mean regardless of GMM modes], 1st most frequent posture [GMM mode with largest area under the curve], 2nd most frequent posture [GMM mode with second largest area], and nearest posture [GMM mode from the real world closest to the mean lumbar sitting angle for each laboratory posture]. The proportion of time spent during sitting and walking in the real world was estimated relative to total activPAL duration. Overall balance performance and spine coordination in sagittal and frontal planes during unstable sitting (laboratory) were measured by calculating (1) seat root mean square (RMS) velocity and displacement and (2) lumbar spine coherence (correlation between L5/S1 and T12/L1 signals) in relation to seat movement in the 0–0.5 and 0.5–1 Hz frequency bands.

### 2.5. Statistical Analysis

Statistical analyses were performed using StataIC 16. *p* < 0.05 was considered statistically significant. To characterise differences within sitting postures, paired t-tests compared the mean lumbar flexion angles between laboratory sitting postures.

To investigate the relationship between mean lumbar angles in the laboratory sitting tasks and each real-world mode (aim 1), we performed pairwise correlations and linear regression analyses with BMI as a covariate. For unstable sitting, whether or not the safety bar was touched during testing was also included as a covariate (4/21 participants touched the bar). Analyses were performed separately for each laboratory sitting posture versus modes of sitting posture in the real world. To investigate the extent to which individuals used the real-world mode that was closest to each laboratory task, we separately plotted the relationship between the difference in angle between the laboratory posture and the nearest real-world posture mode and the relevance of the mode (proportion of time that posture was used).

To determine whether the real-world postures were more related to the posture adopted during unstable sitting than other laboratory sitting postures (aim 2), we compared the correlation/regression coefficients. To investigate whether overall balance performance and/or spine coordination during unstable sitting (laboratory) was related to the proportion of time spent sitting or walking (real world) (aim 3), we performed pairwise correlations and linear regression (covariates—BMI; safety bar touch). The relationship between overall balance performance or spine coordination (unstable sitting) and mean lumbar sagittal angle in this task (aim 4) was investigated using pairwise correlations and linear regression (covariates—BMI; safety bar touch).

## 3. Results

### 3.1. Characteristics of Laboratory Sitting

**Figure 2:**
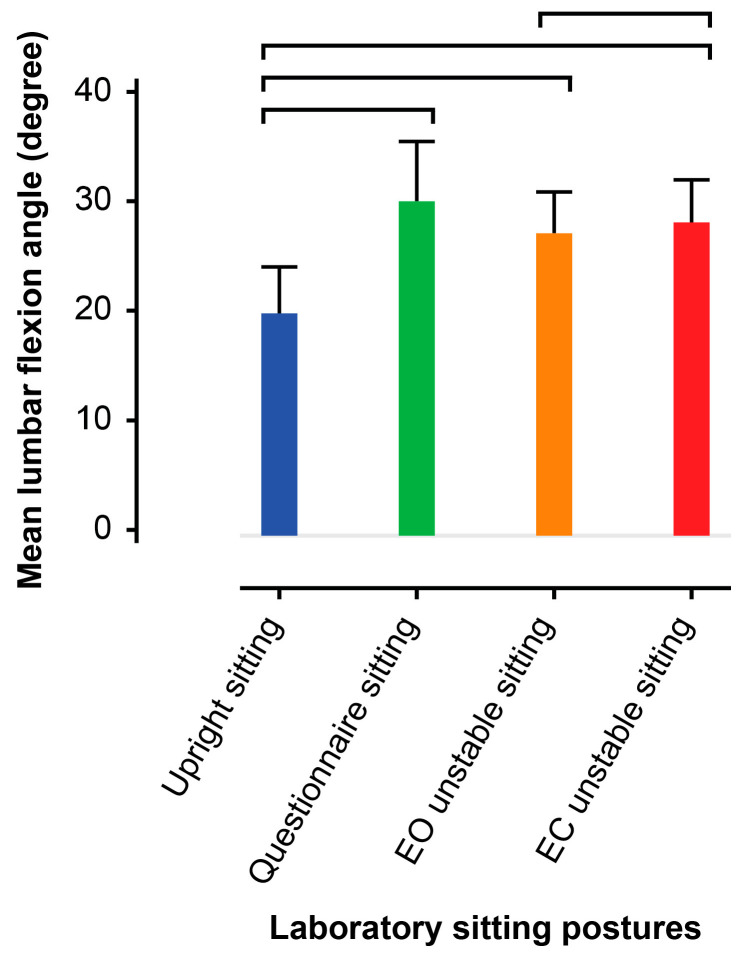
Average angles of laboratory sitting postures, with 0 degrees as the standing upright reference. The analysis compared mean lumbar flexion angles within sitting postures adopted in the laboratory (upright sitting, questionnaire sitting, eyes open [EO] unstable sitting, eyes closed [EC] unstable sitting). Bars indicate a significant difference between pairs of postures. Error bars indicate the 95% confidence intervals.

The average (standard deviation) mean lumbar angles during upright, questionnaire, and unstable sitting (EO and EC) in the laboratory (Figure 2) were 19.8(10.3)°, 30.0(13.3)°, 27.1(9.2)°, and 28.1(9.5)°, respectively. The flexion angle in upright sitting was less (all, *p* < 0.001) than in the other tasks, and the flexion angle during EC unstable sitting was greater (*p* < 0.05) than that with EO.

### 3.2. Relationship Between Laboratory and Real-World Sitting Postures

**Figure 3:**
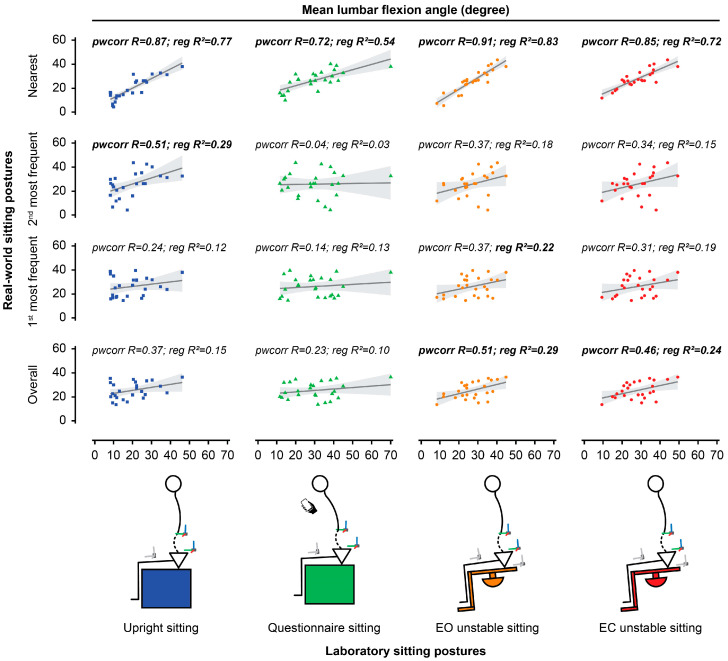
Scatter plots between laboratory and real-world sitting postures. Lumbar spine curvature in sitting is represented by the mean lumbar flexion angle. The analysis investigated the relationship between each sitting posture in the laboratory (*x*-axis: upright sitting, questionnaire sitting, eyes open [EO] unstable sitting, eyes closed [EC] unstable sitting) and sitting postures adopted in the real world (*y*-axis: overall posture, 1st most frequent posture, 2nd most frequent posture, nearest posture). Bold font for pwcorr (pairwise correlation, R = correlation coefficient) or reg (linear regression, R^2^ = determination coefficient) indicates a significant relationship. Shaded grey represents the 95% confidence interval.

The mean lumbar angles during EO (all, *p* < 0.01) and EC unstable sitting (all, *p* < 0.05) in the laboratory were positively associated with the mean lumbar flexion angle adopted during overall real-world sitting. The lumbar angle during upright and questionnaire sitting did not relate to the posture adopted in overall real-world sitting. The mean lumbar flexion angle during EO unstable sitting (*p* < 0.05) was positively associated with that adopted during the real-world 1st most frequent posture mode, whereas the other tasks were not. The mean lumbar flexion angle during upright sitting was positively associated with that adopted during the real-world 2nd most frequent posture mode (all, *p* < 0.05), whereas the other tasks were not. Figure 3 and Table 1 show the correlation/regression results.

**Figure 4:**
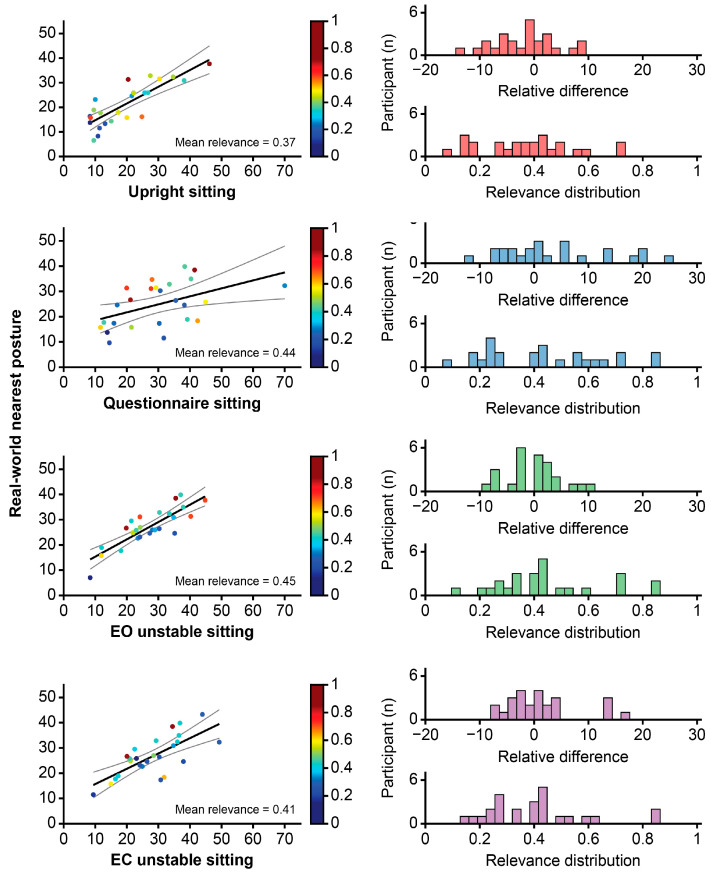
Relationships between laboratory sitting postures and the real-world mode of sitting nearest to that task. Scatter plots (**left column**) show the relationship between laboratory sitting posture (upright sitting, questionnaire sitting, eyes open [EO] unstable sitting, eyes closed [EC] unstable sitting) and posture adopted in the nearest mode of real-world sitting. The distributions of the relevance of these nearest modes and their relative difference in angle (residuals) compared to the laboratory posture are also shown (**right column**). The relevance distribution (**right column**) and dot colour (**left column)** indicate whether the postures adopted in the laboratory and related to the real world are consistently highly relevant (i.e., proportion of overall distribution of postures). The relevance level ranges between 1 (perfect relevance; red) and 0 (no relevance; blue).

The lumbar angle during each laboratory sitting task was also compared to the nearest GMM mode of sitting in the real world for each participant. The mean lumbar sagittal angles in the laboratory sitting tasks were all positively associated with that of the nearest real-world modes (all, *p* < 0.01; Figure 3, Table 1). The mean relevance of the modes nearest to upright, questionnaire, and unstable (EO and EC) sitting was 0.37, 0.44, 0.45, and 0.41, respectively (Figure 4), but was highly variable, ranging between ~0.1 and 0.85.

### 3.3. Relationship Between Performance During Unstable Sitting in the Laboratory and Sedentary/Activity Behaviours in the Real World

**Figure 5:**
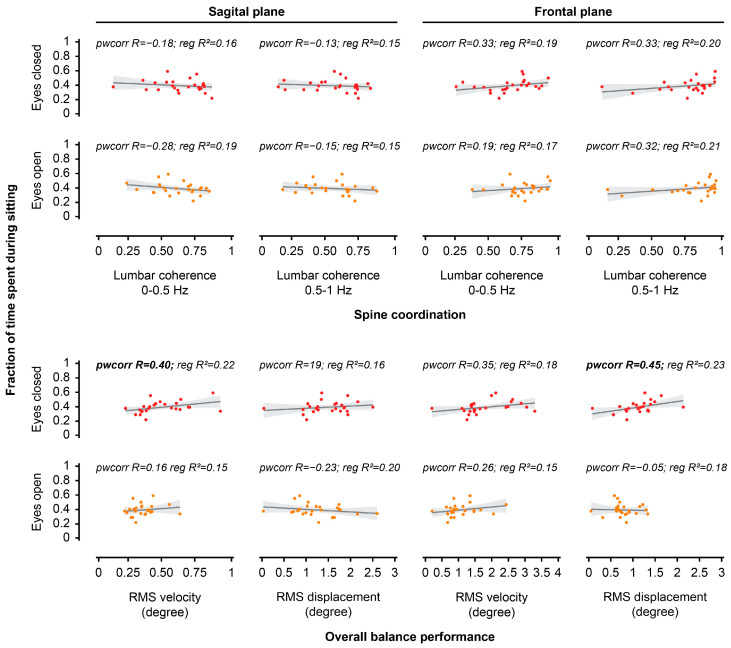
Scatter plots presenting the relationship between overall balance performance or spine coordination in the laboratory and time spent during sitting in the real world. The analysis investigated the relationship between each outcome measure during unstable sitting (**bottom row**: root mean square [RMS] velocity, RMS displacement, **top row**: lumbar-seat coherence) for both visual conditions (eyes open, eyes closed) and planes (sagittal plane, frontal plane) in the laboratory and fraction of time spent during sitting in the real world. Bold font for pwcorr (pairwise correlation, R = correlation coefficient) or reg (linear regression, R^2^ = determination coefficient) indicates a significant relationship.

**Figure 6:**
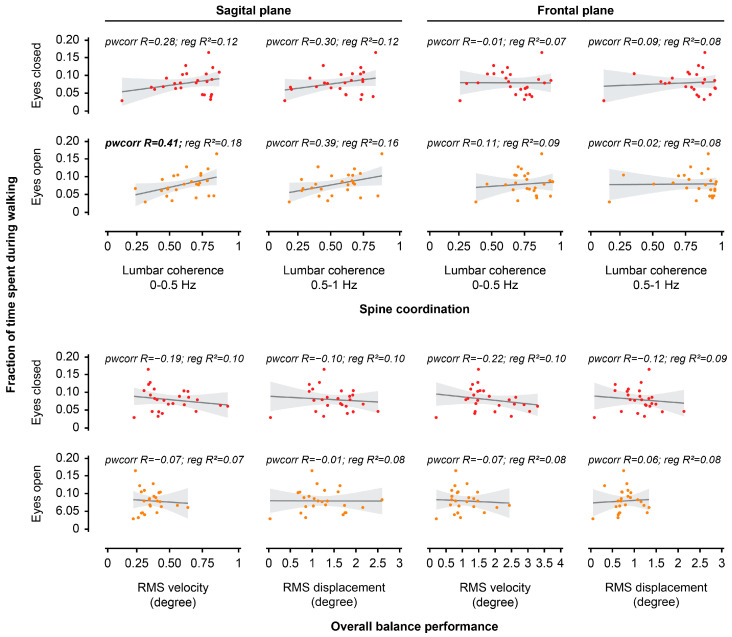
Scatter plots presenting the relationship between overall balance performance or spine coordination in the laboratory and time spent during walking in the real world. The analysis investigated the relationship between each outcome measure during unstable sitting (**bottom row**: root mean square [RMS] velocity, RMS displacement, **top row**: lumbar-seat coherence) for both visual conditions (eyes open, eyes closed) and planes (sagittal plane, frontal plane) in the laboratory and fraction of time spent during walking in the real world. Bold font for pwcorr (pairwise correlation, R = correlation coefficient) or reg (linear regression, R^2^ = determination coefficient) indicates a significant relationship.

Sagittal RMS velocity and frontal RMS displacement during EC unstable sitting were both positively correlated with the proportion of time spent during sitting in the real world (all, *p* < 0.05; Figure 5, Table 2). Sagittal lumbar-seat coherence (at 0–0.5 Hz) during EO unstable sitting was positively correlated with the proportion of time spent during walking in the real world (*p* < 0.05; Figure 6, Table 2).

### 3.4. Relationship Between Spine Posture and Performance During Unstable Sitting

**Figure 7:**
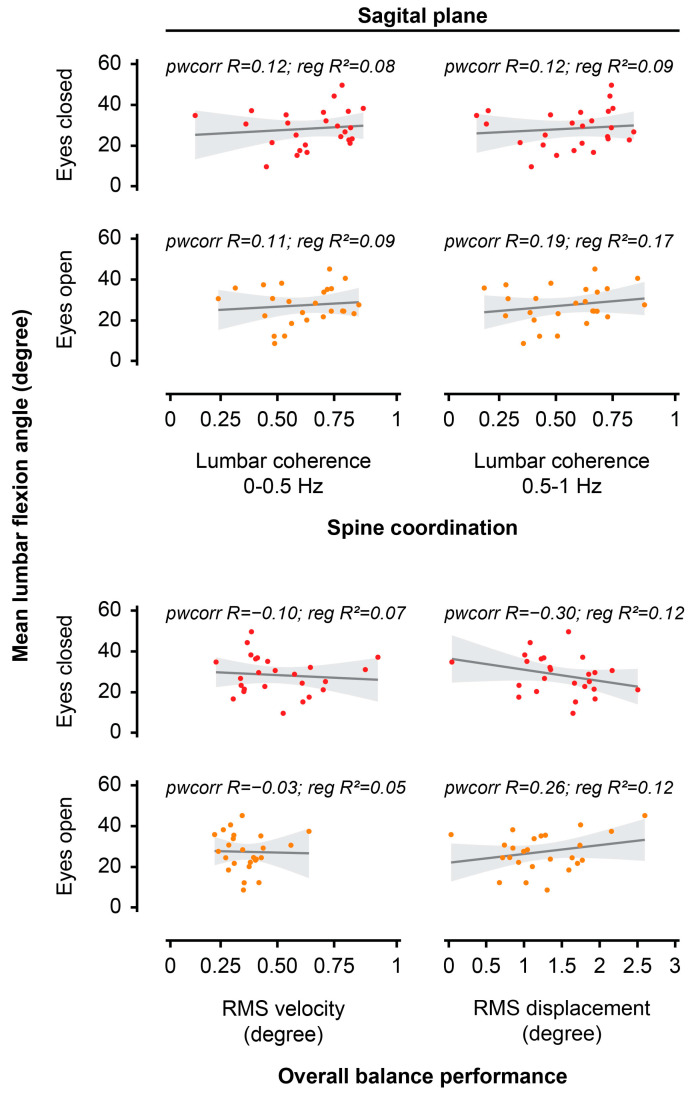
Scatter plots presenting the relationship between spine posture and overall balance performance or spine coordination during unstable sitting. The analysis investigated the relationship between the mean lumbar flexion angle and root mean square (RMS) velocity, RMS displacement, or lumbar-seat coherence in the sagittal plane for each visual condition (eyes open, eyes closed) during unstable sitting in the laboratory.

The mean lumbar flexion angles during EO and EC unstable sitting postures were not associated with RMS velocity, RMS displacement, or lumbar-seat coherence in the sagittal plane during the unstable sitting task (Figure 7, Table 3).

## 4. Discussion

This study has several key observations. First, the posture adopted during unstable sitting in the laboratory was related to the overall and 1st most frequent postures adopted in the real world. Second, the posture adopted during upright sitting in the laboratory related to the real-world 2nd most frequent posture. Third, all laboratory sitting postures were positively associated with at least one mode of sitting (nearest) in the real world, but their relevance was variable. Fourth, participants with less sedentary time exhibited tighter trunk postural control during unstable sitting in the laboratory. Fifth, performance during unstable sitting was not related to the spinal posture adopted during the task.

### 4.1. Relationship Between Laboratory and Real-World Sitting Postures

The angle of lumbar spine curvature in the sagittal plane is commonly used to characterise sitting posture in LBP [2]. In this study, the mean lumbar flexion angles of sitting postures adopted during different laboratory tasks differed in their relationship to that adopted during sitting in the real world. Neither the posture measured during upright sitting nor that during the unconstrained questionnaire sitting task in the laboratory related to either the real-world overall posture or the 1st most common sitting posture. This suggests that static postures adopted in response to specific instructions (e.g., sit upright) or unconstrained tasks in the laboratory differed from postures commonly employed in real-world settings. The significant correlation between posture measured during unstable sitting and the overall or 1st most common sitting posture adopted in the real world implies more dynamic/challenging sitting tasks provide better prediction of the average and most frequent postures adopted in daily life. Although our calculation of GMM modes provides a coarse description of natural sitting posture, this relationship cannot reflect the diversity of sitting behaviours in the real world that depend on the individual and their environment, and it is unlikely that any individual laboratory recording of sitting can capture such complexity. Despite this, evaluation of unstable and upright sitting provides some information on the distribution of postures in the real world.

No prior studies have assessed the behaviour of lumbar spine posture for long periods in the real world, but sitting posture has been measured in the laboratory. One study showed that pain-free individuals adopted a more flexed sitting posture when performing an unconstrained sitting task of using a computer while sitting on a stool [17] but adopted an upright posture when instructed to demonstrate the “ideal” sitting posture (∼10° mean difference of lumbar angle between postures). This corroborates our observation that the spinal posture adopted when asked to sit upright differs from the unconstrained posture to complete a questionnaire.

The close relationship between unstable sitting posture (laboratory) and real-world sitting postures has several plausible explanations. First, the task’s challenging nature might distract attention from spine posture, resulting in adoption of their “typical” spine posture to successfully navigate the task. Second, compared to the other laboratory postures that were either less (upright sitting) or more (questionnaire sitting) flexed than unstable sitting, the lumbar curvature during unstable sitting is mid-range, providing flexibility for adjustment in both directions, with advantages for maintenance of balance. This is supported by previous observations of better performance when a more usual/natural posture is employed in other tasks. For instance, maintenance of balance during standing was more successful in a natural posture than a contrived posture towards the end of range (e.g., stooped posture) [30]. Similar principles may account for greater gait stability when individuals walk at their self-selected (natural) speed rather than at an unusual (e.g., slow speed) speed [31].

### 4.2. Relationship Between Performance During Unstable Sitting in the Laboratory and Real-World Sedentary/Activity Behaviours

Physical activity in the real world was related to equilibrium during unstable sitting in the laboratory. Although a causal relationship cannot be established, we speculate that greater real-world physical activity might be associated with enhanced neuromuscular properties involved in maintaining balance when challenged. Exposure to physical activity might contribute to the refinement of these components through improved coordination, muscular strength and endurance [32,33], proprioception [32], and sensory processing [34].

Several studies have shown that a high level of physical activity (self-reported surveys) is related to better outcomes (e.g., less centre of pressure [CoP] movements) associated with trunk postural control during static [33] and dynamic [33,35] standing balance tasks. These differences are more pronounced with increasing task difficulty, e.g., removal of vision [35], and vestibular manipulation [34]. A study of CoP movements during static (on one leg) standing balance in the laboratory and real-world sitting and activity time (activPAL sensor) for seven days showed those with less real-world sitting and more active time had better laboratory balance performance [36]. In another study, athletic individuals had better balance control when sitting on an unstable seat than non-active individuals [37]. Together with our data, these results suggest active individuals have a greater capacity to dynamically control the spine when challenged.

### 4.3. Relationship Between Spine Posture and Performance During Unstable Sitting

Lumbar posture might modify the challenge of unstable sitting or the capacity to control balance. Spine flexion lowers the body’s centre of mass and reduces the torque required to stabilise the body, as has been shown in standing [38]. However, if the spine is flexed to its end range, its controllability is reduced because there is no further spine flexion available to compensate for balance challenges in that direction. We found that lumbar spine posture during unstable sitting was not associated with overall balance performance or spine coordination. The mid-range spine posture adopted in this task appears to balance the trade-offs between these two extremes. The absence of associations across all participants suggests that optimal lumbar posture for unstable sitting is specific for each person (perhaps due to flexibility, strength, or other factors).

### 4.4. Methodological Considerations

Several methodological issues require consideration. First, wearable motion sensors do not directly estimate the anatomical angles of the spine but instead estimate the angle of the spine relative to a reference value. Second, some participants were excluded due to device errors (e.g., error in data offload) or data errors (e.g., magnetic interference). Third, measurements of lumbar spine posture might be affected by skin or soft tissue movement relative to the spine, which can cause errors in estimating the true spinal angle or posture. Fourth, the relatively small sample size of healthy, pain-free participants may limit statistical power and the generalisability of the findings to broader populations, particularly individuals with LBP or those with varying ages or occupational demands. Future studies with larger samples, based on appropriate power calculations, and involving more diverse clinical populations could further strengthen the external validity and clinical applicability of these findings. Fifth, the 48 h monitoring period may not fully capture variations in sitting behaviour across workdays, weekends, or other daily contexts. Although this duration was selected to optimise participant adherence and data quality, longer monitoring periods in the real world (e.g., 5–7 days) would likely provide a more comprehensive reflection of postural behaviour and improve ecological validity. Sixth, although the GMM approach provided a practical method to characterise individual variability in real-world lumbar spine sitting postures, the number of modes was determined using the Calinski–Harabasz criterion and limited to four to reduce overfitting and maintain physiologically interpretable posture clusters. Preliminary data inspection showed that adding more modes did not meaningfully improve model performance. While this approach has not been formally validated for lumbar sitting posture data, it was suitable for the exploratory purpose of this study. Seventh, as this was an exploratory analysis involving several independent regression models aligned with distinct research aims, formal correction for multiple comparisons was not applied. Instead, emphasis was placed on the magnitude and consistency of effects across related analyses. While this approach may increase the likelihood of Type I error, it was considered appropriate for an exploratory study aiming to identify potential relationships to guide future work. Eighth, some associations were near the conventional significance threshold and should, therefore, be interpreted cautiously, particularly given the sample size and exploratory nature of the analyses.

### 4.5. Clinical Implications

The present findings indicate that dynamic sitting tasks, such as unstable sitting, provide a more representative measure of lumbar spine posture than static instructed positions in the real world. In this study, unstable sitting in the laboratory was related to the overall and 1st most frequent real-world sitting postures, whereas upright sitting was related only to the 2nd most frequent real-world sitting posture. These results suggest that clinical and ergonomic assessments could incorporate tasks that challenge trunk postural control rather than relying solely on an instructed upright sitting position. Although unstable sitting correlated most closely with real-world postures, it remains an artificial laboratory paradigm and should be interpreted as an ecological surrogate that reflects the dynamic control strategies and mid-range lumbar angles commonly adopted during everyday sitting rather than a direct replication of free-living posture. The absence of an association between the mean lumbar flexion angle during unstable sitting and measures of balance performance or spine coordination indicates that no single lumbar angle should be considered an optimal clinical target. From a clinical and ergonomic perspective, the unstable sitting task may serve as a useful proxy for identifying individuals who demonstrate limited postural adaptability or a tendency to adopt end-range postures, both of which may increase spinal tissue loading during prolonged sitting. When combined with extended wearable monitoring, such dynamic laboratory tasks can complement real-world assessment and provide a more comprehensive understanding of sitting behaviour. Future research involving individuals with LBP, together with longer-term monitoring in natural environments, is required to confirm these findings and to establish their translational relevance for ergonomic assessment and rehabilitation practice.

## 5. Conclusions

This study suggests that spine posture adopted in a laboratory unstable sitting task is more closely related to real-world lumbar spine postures than other “static” laboratory-based sitting tasks. The association between unstable sitting and activity in daily life provides some insight into factors related to successful trunk postural control. This study provides a foundation to consider the orientation and kinematics of the lumbar spine in free-living unconstrained settings in musculoskeletal conditions such as LBP.

## Figures and Tables

**Table 1:** Relationship between laboratory and real-world sitting postures.

Outcome	Pairwise Correlation			Linear Regression Analysis		
	Coef. ^a^	95% CI	*p*-Value	Coef. ^b^	95% CI	*p*-Value
Real world: overall posture						
Laboratory:						
Upright sitting	0.37	−0.03, 0.67	0.067	0.25	−0.03, 0.54	0.074
Questionnaire sitting	0.23	−0.18, 0.57	0.267	0.18	−0.08, 0.43	0.167
EO unstable sitting	0.51	0.14, 0.75	0.009	0.41	0.12, 0.70	**0.008**
EC unstable sitting	0.46	0.09, 0.73	0.019	0.36	0.07, 0.66	**0.018**
Real world: 1st most frequent posture						
Laboratory:						
Upright sitting	0.24	−0.17, 0.58	0.251	0.20	−0.14, 0.54	0.232
Questionnaire sitting	0.14	−0.27, 0.51	0.492	0.20	−0.10, 0.49	0.177
EO unstable sitting	0.37	−0.04, 0.67	0.073	0.37	0.01, 0.73	**0.043**
EC unstable sitting	0.31	−0.10, 0.63	0.132	0.32	−0.04, 0.68	0.078
Real world: 2nd most frequent posture						
Laboratory:						
Upright sitting	0.51	0.14, 0.75	0.010	0.52	0.13, 0.90	**0.011**
Questionnaire sitting	0.04	−0.37, 0.42	0.867	0.07	−0.32, 0.46	0.696
EO unstable sitting	0.36	−0.04, 0.66	0.073	0.45	−0.02, 0.92	0.060
EC unstable sitting	0.34	−0.06, 0.65	0.096	0.40	−0.06, 0.86	0.084
Real world: nearest posture						
Laboratory:						
Upright sitting	0.87	0.73, 0.94	<0.001	0.80	0.60, 1.00	**<0.001**
Questionnaire sitting	0.72	0.46, 0.87	<0.001	0.41	0.20, 0.62	**0.001**
EO unstable sitting	0.91	0.80, 0.96	<0.001	0.96	0.76, 1.17	**<0.001**
EC unstable sitting	0.85	0.68, 0.93	<0.001	0.68	0.48, 0.88	**<0.001**

Abbreviations: Coef., coefficient; CI, confidence interval; EO, eyes open; EC, eyes closed. Statistics: Two different statistical analyses (pairwise correlations and linear regression) were performed to investigate the relationship between variables for each comparison (row) in this table. *p*-values of statistically significant correlations/regressions (*p* < 0.05) are printed bold. ^a^ correlation coefficient. ^b^ regression coefficient.

**Table 2:** Relationship between performance during unstable sitting in the laboratory and sedentary/activity behaviours in the real world.

Outcome	Pairwise Correlation			Linear Regression Analysis		
	Coef. ^a^	95% CI	*p*-Value	Coef. ^b^	95% CI	*p*-Value
Real-world time spent during sitting						
Laboratory—overall balance performance:						
Sagittal RMS velocity—EO	0.16	−0.25, 0.52	0.447	0.02	−0.37, 0.41	0.904
Sagittal RMS velocity—EC	0.40	0.00, 0.68	**0.049**	0.14	−0.06, 0.33	0.164
Sagittal RMS displacement—EO	−0.23	−0.58, 0.18	0.261	−0.04	−0.10, 0.03	0.238
Sagittal RMS displacement—EC	0.19	−0.22, 0.55	0.353	0.02	−0.05, 0.09	0.566
Frontal RMS velocity—EO	0.26	−0.15, 0.60	0.208	0.01	−0.08, 0.10	0.818
Frontal RMS velocity—EC	0.35	−0.06, 0.65	0.089	0.03	−0.03, 0.08	0.349
Frontal RMS displacement—EO	−0.05	−0.44, 0.35	0.813	−0.05	−0.18, 0.07	0.368
Frontal RMS displacement—EC	0.45	0.06, 0.72	**0.025**	0.07	−0.03, 0.16	0.148
Laboratory—spine coordination:						
Sagittal lumbar coherence (0–0.5 Hz)—EO	−0.28	−0.61, 0.13	0.173	−0.12	−0.36, 0.11	0.274
Sagittal lumbar coherence (0–0.5 Hz)—EC	−0.18	−0.54, 0.23	0.384	−0.05	−0.25, 0.14	0.568
Sagittal lumbar coherence (0.5–1 Hz)—EO	−0.15	−0.52, 0.26	0.463	−0.05	−0.26, 0.17	0.650
Sagittal lumbar coherence (0.5–1 Hz)—EC	−0.13	−0.50, 0.28	0.525	−0.02	−0.21, 0.17	0.843
Frontal lumbar coherence (0–0.5 Hz)—EO	0.19	−0.22, 0.55	0.353	0.09	−0.15, 0.33	0.458
Frontal lumbar coherence (0–0.5 Hz)—EC	0.33	−0.07, 0.64	0.106	0.10	−0.10, 0.30	0.300
Frontal lumbar coherence (0.5–1 Hz)—EO	0.32	−0.08, 0.64	0.117	0.10	−0.06, 0.25	0.222
Frontal lumbar coherence (0.5–1 Hz)—EC	0.33	−0.08, 0.64	0.112	0.10	−0.07, 0.27	0.232
Real-world time spent during walking						
Laboratory—overall balance performance:						
Sagittal RMS velocity—EO	−0.07	−0.45, 0.33	0.733	−0.00	−0.16, 0.16	0.977
Sagittal RMS velocity—EC	−0.19	−0.54, 0.22	0.364	−0.03	−0.11, 0.06	0.492
Sagittal RMS displacement—EO	−0.01	−0.40, 0.39	0.980	−0.00	−0.03, 0.02	0.821
Sagittal RMS displacement—EC	−0.10	−0.48, 0.31	0.628	−0.01	−0.04, 0.02	0.468
Frontal RMS velocity—EO	−0.07	−0.45, 0.34	0.754	−0.00	−0.04, 0.04	0.966
Frontal RMS velocity—EC	−0.22	−0.57, 0.19	0.291	−0.01	−0.03, 0.01	0.451
Frontal RMS displacement—EO	0.06	−0.34, 0.45	0.762	0.01	−0.04, 0.06	0.676
Frontal RMS displacement—EC	−0.12	−0.49, 0.29	0.564	−0.01	−0.05, 0.03	0.567
Laboratory—spine coordination:						
Sagittal lumbar coherence (0–0.5 Hz)—EO	0.41	0.02, 0.69	**0.042**	0.07	−0.02, 0.17	0.119
Sagittal lumbar coherence (0–0.5 Hz)—EC	0.28	−0.13, 0.61	0.174	0.04	−0.04, 0.12	0.301
Sagittal lumbar coherence (0.5–1 Hz)—EO	0.39	−0.01, 0.68	0.056	0.06	−0.03, 0.14	0.168
Sagittal lumbar coherence (0.5–1 Hz)—EC	0.30	−0.11, 0.62	0.151	0.04	−0.04, 0.11	0.325
Frontal lumbar coherence (0–0.5 Hz)—EO	0.11	−0.30, 0.48	0.608	0.03	−0.07, 0.13	0.578
Frontal lumbar coherence (0–0.5 Hz)—EC	−0.01	−0.40, 0.39	0.979	0.00	−0.08, 0.08	0.998
Frontal lumbar coherence (0.5–1 Hz)—EO	0.02	−0.38, 0.41	0.939	0.00	−0.07, 0.07	0.963
Frontal lumbar coherence (0.5–1 Hz)—EC	0.09	−0.32, 0.47	0.666	0.01	−0.06, 0.09	0.712

Abbreviations: Coef., coefficient; CI, confidence interval; RMS, root mean square; EO, eyes open; EC, eyes closed. Statistics: Two different statistical analyses (pairwise correlations and linear regression) were performed to investigate the relationship between variables for each comparison (row) in this table. *p*-values of statistically significant correlations/regressions (*p* < 0.05) are printed bold. ^a^ correlation coefficient. ^b^ regression coefficient.

**Table 3:** Relationship between spine posture and performance during unstable sitting.

Outcome	Pairwise Correlation			Linear Regression Analysis		
	Coef. ^a^	95% CI	*p*-Value	Coef. ^b^	95% CI	*p*-Value
Mean lumbar flexion angle						
Overall balance performance:						
RMS velocity—EO	−0.03	−0.42, 0.37	0.898	−10.73	−57.11, 35.65	0.635
RMS velocity—EC	−0.10	−0.48, 0.31	0.640	−9.00	−33.87, 15.88	0.460
RMS displacement—EO	0.26	−0.16, 0.59	0.218	4.89	−2.42, 12.19	0.179
RMS displacement—EC	−0.30	−0.62, 0.11	0.149	−5.50	−13.78, 2.78	0.182
Spine coordination:						
Lumbar coherence (0–0.5 Hz)—EO	0.11	−0.30, 0.48	0.603	15.02	−12.64, 42.68	0.272
Lumbar coherence (0–0.5 Hz)—EC	0.12	−0.29, 0.49	0.579	9.92	−13.67, 33.51	0.392
Lumbar coherence (0.5–1 Hz)—EO	0.19	−0.22, 0.55	0.351	20.55	−3.45, 44.55	0.089
Lumbar coherence (0.5–1 Hz)—EC	0.12	−0.29, 0.49	0.577	11.97	−10.93, 34.86	0.289

**Abbreviations**: Coef., coefficient; CI, confidence interval; RMS, root mean square; EO, eyes open; EC, eyes closed. **Statistics**: Two different statistical analyses (pairwise correlations and linear regression) were performed to investigate the relationship between variables for each comparison (row) in this table. ^a^ correlation coefficient. ^b^ regression coefficient.

## Data Availability

The original contributions presented in this study are included in the article; further inquiries can be directed at the corresponding author.

## Supplementary Materials

The following supporting information can be downloaded at: https://www.mdpi.com/article/10.3390/jcm14217518/s1, File S1: Information about data synchronisation, segmentation, and exclusion [39].

## Abbreviations

The following abbreviations are used in this manuscript:

LBP	Low back pain
BMI	Body mass index
L5/S1	Lumbosacral junction
T12/L1	Thoracolumbar junction
EO	Eyes open
EC	Eyes closed
GMMs	Gaussian Mixture Models
RMS	Root mean square
pwcorr	Pairwise correlation
R^2^	Determination coefficient

## References

[1] Owen N., Healy G.N., Matthews C.E., Dunstan D.W. (2010). Too much sitting: The population health science of sedentary behavior. Exerc. Sport Sci. Rev..

[2] Mörl F., Bradl I. (2013). Lumbar posture and muscular activity while sitting during office work. J. Electromyogr. Kinesiol..

[3] Soares C., Shimano S.G.N., Marcacine P.R., Fernandes L.F.R.M., de Castro L.L.P.T., de Walsh I.A.P. (2023). Ergonomic interventions for work in a sitting position: An integrative review. Rev. Bras. Med. Trab..

[4] Kim S., Lee I., Kang S.H., Jin S. (2023). Significance of lower body postures in chair design. Hum. Factors.

[5] Alshehri M.A., Alzahrani H., van den Hoorn W., Klyne D.M., Vette A.H., Hendershot B.D., Roberts B.W., Larivière C., Barbado D., Vera-Garcia F.J. (2024). Trunk postural control during unstable sitting among individuals with and without low back pain: A systematic review with an individual participant data meta-analysis. PLoS ONE.

[6] Claeys K., Brumagne S., Deklerck J., Vanderhaeghen J., Dankaerts W. (2016). Sagittal evaluation of usual standing and sitting spinal posture. J. Bodyw. Mov. Ther..

[7] Kwon Y., Kim J.W., Heo J.H., Jeon H.M., Choi E.B., Eom G.M. (2018). The effect of sitting posture on the loads at cervico-thoracic and lumbosacral joints. Technol. Health Care.

[8] In T.S., Jung J.H., Jung K.S., Cho H.Y. (2021). Spinal and pelvic alignment of sitting posture associated with smartphone use in adolescents with low back pain. Int. J. Environ. Res. Public Health.

[9] Dankaerts W., O’Sullivan P., Burnett A., Straker L. (2006). Differences in sitting postures are associated with nonspecific chronic low back pain disorders when patients are subclassified. Spine.

[10] Misir A., Kizkapan T.B., Tas S.K., Yildiz K.I., Ozcamdalli M., Yetis M. (2019). Lumbar spine posture and spinopelvic parameters change in various standing and sitting postures. Eur. Spine J..

[11] Alshehri M.A., van den Hoorn W., Klyne D.M., Hodges P.W. (2024). Coordination of hip and spine in individuals with acute low back pain during unstable sitting. Spine J..

[12] Alshehri M.A., van den Hoorn W., Klyne D.M., van Dieën J.H., Cholewicki J., Hodges P.W. (2024). Poor lumbar spine coordination in acute low back pain predicts persistent long-term pain and disability. Eur. Spine J..

[13] Hodges P.W., Danneels L. (2019). Changes in structure and function of the back muscles in low back pain: Different time points, observations, and mechanisms. J. Orthop. Sports Phys. Ther..

[14] Cholewicki J., Silfies S.P., Shah R.A., Greene H.S., Reeves N.P., Alvi K., Goldberg B. (2005). Delayed trunk muscle reflex responses increase the risk of low back injuries. Spine.

[15] Coenen P., Kingma I., Boot C.R., Twisk J.W., Bongers P.M., van Dieën J.H. (2013). Cumulative low back load at work as a risk factor of low back pain: A prospective cohort study. J. Occup. Rehabil..

[16] Hodges P.W., van den Hoorn W. (2022). A vision for the future of wearable sensors in spine care and its challenges: Narrative review. J. Spine Surg..

[17] Claus A.P., Hides J.A., Moseley G.L., Hodges P.W. (2016). Thoracic and lumbar posture behaviour in sitting tasks and standing: Progressing the biomechanics from observations to measurements. Appl. Ergon..

[18] Kuo Y.L., Huang K.Y., Kao C.Y., Tsai Y.J. (2021). Sitting posture during prolonged computer typing with and without a wearable biofeedback sensor. Int. J. Environ. Res. Public Health.

[19] Cholewicki J., Polzhofer G., Radebold A. (2000). Postural control of trunk during unstable sitting. J. Biomech..

[20] Alshehri M.A., van den Hoorn W., Klyne D., Hodges P.W. (2021). Coordination of hip and spine to maintain equilibrium in unstable sitting revealed by spectral analysis. J. Neurophysiol..

[21] Roberts B.W., Gholibeigian F., Lewicke J., Vette A.H. (2020). Spatial and temporal relation of kinematics and muscle activity during unstable sitting. J. Electromyogr. Kinesiol..

[22] Simpson L., Maharaj M.M., Mobbs R.J. (2019). The role of wearables in spinal posture analysis: A systematic review. BMC Musculoskelet. Disord..

[23] Papi E., Koh W.S., McGregor A.H. (2017). Wearable technology for spine movement assessment: A systematic review. J. Biomech..

[24] Larivière C., Mecheri H., Shahvarpour A., Gagnon D., Shirazi-Adl A. (2013). Criterion validity and between-day reliability of an inertial-sensor-based trunk postural stability test during unstable sitting. J. Electromyogr. Kinesiol..

[25] Mjøsund H.L., Boyle E., Kjaer P., Mieritz R.M., Skallgård T., Kent P. (2017). Clinically acceptable agreement between the ViMove wireless motion sensor system and the Vicon motion capture system when measuring lumbar region inclination motion in the sagittal and coronal planes. BMC Musculoskelet. Disord..

[26] Khurelbaatar T., Kim K., Lee S., Kim Y.H. (2015). Consistent accuracy in whole-body joint kinetics during gait using wearable inertial motion sensors and in-shoe pressure sensors. Gait Posture.

[27] Kos A., Tomažič S., Umek A. (2016). Evaluation of smartphone inertial sensor performance for cross-platform mobile applications. Sensors.

[28] Riddick R., Smits E., Faber G., Shearwin C., Hodges P., van den Hoorn W. (2023). Estimation of human spine orientation with inertial measurement units (IMU) at low sampling rate: How low can we go?. J. Biomech..

[29] Cotter B.D., Nairn B.C., Drake J.D. (2014). Should a standing or seated reference posture be used when normalizing seated spine kinematics?. J. Biomech..

[30] Jacobs J.V., Dimitrova D.M., Nutt J.G., Horak F.B. (2005). Can stooped posture explain multidirectional postural instability in patients with Parkinson’s disease?. Exp. Brain Res..

[31] Beauchet O., Annweiler C., Lecordroch Y., Allali G., Dubost V., Herrmann F.R., Kressig R.W. (2009). Walking speed-related changes in stride time variability: Effects of decreased speed. J. Neuroeng. Rehabil..

[32] Zhu W., Li Y., Wang B., Zhao C., Wu T., Liu T., Sun F. (2021). Objectively measured physical activity is associated with static balance in young adults. Int. J. Environ. Res. Public Health.

[33] Alsufiany M.B., Lohman E.B., Daher N.S., Gang G.R., Shallan A.I., Jaber H.M. (2020). Non-specific chronic low back pain and physical activity: A comparison of postural control and hip muscle isometric strength: A cross-sectional study. Medicine.

[34] Maitre J., Paillard T. (2016). Postural effects of vestibular manipulation depend on the physical activity status. PLoS ONE.

[35] Onofrei R.R., Amaricai E. (2022). Postural balance in relation with vision and physical activity in healthy young adults. Int. J. Environ. Res. Public Health.

[36] Cooper R., Stamatakis E., Hamer M. (2020). Associations of sitting and physical activity with grip strength and balance in mid-life: 1970 British Cohort Study. Scand. J. Med. Sci. Sports.

[37] Glofcheskie G.O., Brown S.H.M. (2017). Athletic background is related to superior trunk proprioceptive ability, postural control, and neuromuscular responses to sudden perturbations. Hum. Mov. Sci..

[38] Kim S.S., Lim K., Choi W.J. (2021). Effect on the center of pressure of vision, floor condition, and the height of center of mass during quiet standing. Phys. Ther. Korea.

[39] Ligorio G., Sabatini A.M. (2016). Dealing with magnetic disturbances in human motion capture: A survey of techniques. Micromachines.

