# Supplementary material for: Exploring Lumbar Spine Posture and Movement in Sitting: A Comparison Between Laboratory and Real-World Measures
Source: J Clin Med. 2025 Oct 23;14(21):7518. doi: 10.3390/jcm14217518 (PMC12608425; doi:10.3390/jcm14217518)
Supplement: Supplementary file 1 [file jcm-14-07518-s001.zip › jcm-3911172-supplementary.pdf]

# Supplementary material S1

## Data processing

### *Data synchronisation*

Initial synchronisation was achieved by cross-correlating the accelerometer signal of all sensors (dorsaVi and activPAL) while they were fixed (taped) to a board during a series of calibration movements prior to attachment to the participant. Although this method was sufficient for the ~1 hour laboratory session, some "drift" in the synchronisation of the sensors was observed over the 48 hours of real-world testing. We computed this lag by cross-correlating between each sensor every hour. The slope of a linear fit between lag and time was used to estimate the difference in sample rate between the two sensors. With one sensor used as the reference, all other sensors were resampled using this estimated difference in sample rate.

### *Data segmentation*

To extract the periods of time during which participants performed specific tasks, a combination of automatic and manual processing was performed. For laboratory testing, flags were triggered by the assessor to denote the start and end of each task using the dorsaVi Research Package Software (version RD.A574.F). Participants were also asked to hop five times prior to certain task. An algorithm was then used to identify any period in which the participant hopped based on the magnitude of acceleration. For real-world data, we used labels from activPAL's analysis to detect periods of sitting, standing, stepping, laying, and seated transport. These labels were provided at a 1 s resolution and were up-sampled to 20 Hz to match the accelerometer of the dorsaVi sensors.

### *Data exclusion*

Because magnetic interference is common (Ligorio and Sabatini, 2016) (e.g., when driving in a car), we implemented an algorithm for its detection. This algorithm detects any substantial changes in the expected magnitude of the Earth's magnetic field that would indicate

interference. Data captured during, including 30 seconds before and after, the detection of magnetic disturbances were excluded. We also excluded five minutes of data prior to or after any detected seated (moving) transport as the associated large accelerations make orientation estimation unreliable.

## References


Ligorio, G., Sabatini, A.M., 2016. Dealing with magnetic disturbances in human motion capture: A survey of techniques. Micromachines 7, 43.
